# Homeostatic Plasticity Achieved by Incorporation of Random Fluctuations and Soft-Bounded Hebbian Plasticity in Excitatory Synapses

**DOI:** 10.3389/fncir.2016.00042

**Published:** 2016-06-01

**Authors:** Takashi Matsubara, Kuniaki Uehara

**Affiliations:** Computational Intelligence, Fundamentals of Computational Science, Department of Computational Science, Graduate School of System informatics, Kobe UniversityKobe-shi, Japan

**Keywords:** random fluctuation, homeostatic plasticity, spike-timing dependent plasticity, synaptic competition, neural network

## Abstract

Homeostatic plasticity is considered to maintain activity in neuronal circuits within a functional range. In the absence of homeostatic plasticity neuronal activity is prone to be destabilized because Hebbian plasticity mechanisms induce positive feedback change. Several studies on homeostatic plasticity assumed the existence of a process for monitoring neuronal activity on a time scale of hours and adjusting synaptic efficacy by scaling up and down. However, the underlying mechanism still remains unclear. Excitatory synaptic efficacy is associated with the size of the dendritic spine, and dendritic spine size fluctuates even after neuronal activity is silenced. These fluctuations could be a non-Hebbian form of synaptic plasticity that serves such a homeostatic function. This study proposed and analyzed a synaptic plasticity model incorporating random fluctuations and soft-bounded Hebbian plasticity at excitatory synapses, and found that the proposed model can prevent excessive changes in neuronal activity by scaling synaptic efficacy up and down. Soft-bounded Hebbian plasticity suppresses strong synapses, thereby scaling synapses down and preventing runaway excitation. Random fluctuations diffuse synaptic efficacy, thereby scaling synapses up and preventing neurons from falling silent. The proposed model acts as a form of homeostatic plasticity, regardless of neuronal activity monitoring.

## 1. Introduction

Synaptic modifications are the fundamental feature for brain development and cognitive processes such as attention, perception, and memory (Bliss and Lømo, 1973; Martin et al., 2000; Whitlock et al., 2006; Nabavi et al., 2014). Synaptic efficacy is adjusted mainly by the process termed as *Hebbian plasticity*, which depends on the correlation between the activities of the pre- and post-synaptic neurons (Löwel and Singer, 1992; Katz and Shatz, 1996). Traditional Hebbian plasticity and its temporally asymmetric form, *spike-timing dependent plasticity* (STDP) (Markram et al., 1997; Bi and Poo, 1998; Sjöström et al., 2001), are positive feedback processes that are inherently prone to destabilize the neuronal activity (Rochester et al., 1956; von der Malsburg, 1973; Oja, 1982; Miller and MacKay, 1994; Miller, 1996). Synaptic potentiation due to a correlation between activities of pre- and post-synaptic neurons further increases the correlation, leading to further potentiation. Once depressed synapse no longer leads to the correlated activities of neurons, leading to further depression and becoming silent. To prevent these instability issues, various constraints on synaptic efficacy have been introduced; limit in the total sum of synaptic efficacy projecting to one neuron (von der Malsburg, 1973; Effenberger et al., 2015), decay in synaptic efficacy (Oja, 1982; Miller and MacKay, 1994), and negative feedback process (Bienenstock et al., 1982; Bear et al., 1987; Miller, 1996). Moreover, so-called “soft-bounded” STDP models have been proposed (van Rossum et al., 2000; Gütig et al., 2003; Morrison et al., 2007; Gilson and Fukai, 2011), in which depression at a strong synapse is much larger than that at a weak synapse. Soft-bounded STDP models provide an equilibrium at which potentiation and depression are balanced and prevent runaway potentiation or depression. Such dependence on efficacy is supported by evidences from biological studies (Bi and Poo, 1998). However, stability of synaptic efficacy does not always indicate stability of neuronal activity. The ranges of parameter values with which soft-bounded STDP models provide stability of neuronal activity are limited (Morrison et al., 2007; Gilson and Fukai, 2011; Yger and Gilson, 2015). Another negative feedback process, *homeostatic plasticity*, has also been proposed to maintain neuronal activity within a functional range (van Rossum et al., 2000; Turrigiano, 2008; Watt and Desai, 2010; Zenke et al., 2013; Toyoizumi et al., 2014). It is typically modeled as a proportional or proportional-integral controller that monitors post-synaptic neuronal activity on a time scale of hours, scales synaptic efficacy up or down, and stabilizes post-synaptic neuronal activity. Several neurobiological studies have confirmed the existence of homeostatic plasticity in mammalian cortex and hippocampus (Rutherford et al., 1998; Turrigiano et al., 1998; Turrigiano, 1999, 2008; Burrone et al., 2002; Ibata et al., 2008; Keck et al., 2013; Vitureira and Goda, 2013; Félix-Oliveira et al., 2014). After neuronal activity is decreased or silenced by drugs or lesions in sensory organs, synapses are potentiated on average, despite a decrease in correlated activity. Conversely, after neuronal activity is increased by drugs, synapses are depressed on average. Moreover, the normalized distribution of excitatory synaptic efficacy is almost similar before and after the change in neuronal activity. Therefore, this type of homeostatic plasticity is called *synaptic scaling* or *activity-dependent scaling*^1^. Synaptic scaling is considered to consist of two distinct underlying mechanisms; *scaling up* (potentiation against low neuronal activity.) and *scaling down* (depression against high neuronal activity). Neuronal activity converges to the rate at which scaling up and scaling down are balanced. However, the mechanism underlying synaptic scaling still remains unclear.

Other studies have identified activity-independent (non-Hebbian) excitatory synaptic modifications called *intrinsic fluctuations* (Yasumatsu et al., 2008; Kasai et al., 2010; Loewenstein et al., 2011; Statman et al., 2014); the size of a post-synaptic element, dendritic spine, fluctuates even after neuronal activity is silenced. Because the efficacy of an excitatory synapse in the mammalian central nervous system is dependent on the corresponding dendritic spine size (Matsuzaki et al., 2001, 2004; Kasai et al., 2003; Kopec et al., 2006; Wang et al., 2007; Yuste, 2010; Keck et al., 2013), it is possible that these intrinsic fluctuations serve homeostatic plasticity mechanism. However, the intrinsic fluctuations are thought to induce change with zero expected value as opposed to synaptic scaling.

This paper proposes an excitatory synaptic plasticity model incorporating intrinsic fluctuations (Yasumatsu et al., 2008) and soft-bounded STDP model (van Rossum et al., 2000), and demonstrates a possible role for these intrinsic fluctuations in scaling of synaptic efficacy to prevent destabilization of neuronal activity. The proposed model demonstrated the robust stability of post-synaptic firing rate in response to change in pre-synaptic firing rate. The distribution of synaptic efficacy was scaled multiplicatively with varying pre-synaptic firing rate. After neuronal activity was silenced, synapses were scaled up. Intrinsic fluctuations also contributed to synaptic competition (Miller, 1996; Song et al., 2000; van Rossum et al., 2000). These numerical experiments and corresponding theoretical analyses indicate that intrinsic fluctuations achieve scaling up of synapses and soft-bounded STDP model achieves scaling down of synapses. As a result, synaptic plasticity model incorporating intrinsic fluctuations and soft-bounded STDP model acts as a form of homeostatic plasticity without monitoring neuronal activity.

## 2. Models and methods

### 2.1. Intrinsic fluctuations

Recent studies have reported non-Hebbian excitatory synaptic modifications called *intrinsic fluctuations* (Yasumatsu et al., 2008; Kasai et al., 2010; Loewenstein et al., 2011; Statman et al., 2014). Dendritic spines change size even after neuronal activity is silenced by drugs. Because spine size is strongly related to synaptic efficacy (Matsuzaki et al., 2001, 2004; Kasai et al., 2003; Kopec et al., 2006; Wang et al., 2007; Yuste, 2010; Keck et al., 2013), these intrinsic fluctuations are thought to influence synaptic efficacy independently from pre-synaptic firing and post-synaptic firing. According to the original studies, intrinsic fluctuations follow the stochastic differential equation as follows:

(1) $$dW_{t}=(SW_{t}+s)dB_{t},$$

where *W*_*t*_ denotes the excitatory synaptic weight *W* at time *t*, and *B*_*t*_ is the standard Wiener process (i.e., the continuous-time stochastic process with zero mean and unit variance per unit time). Equation (1) implies that the expected change E[*W*_*t*_ − *W*_0_] in synaptic weight *W* is zero. However, in this study, the synaptic weight was bounded at zero to prevent a negative weight; thus, the expected change E[*W*_*t*_ − *W*_0_] at near-zero synaptic weight was positive. Yasumatsu et al. (2008) estimated the parameter values *S* = 0.2 and *s* = 0.01 μm^3^ per day. This study empirically employed parameter values of *S* = 0.2 and *s* = 7000 pS per day.

### 2.2. Spike-timing dependent plasticity

Spike-timing dependent plasticity (STDP) is a temporally asymmetric form of Hebbian plasticity that allows both depression and potentiation depending on the temporal relationship between pre-synaptic firing and post-synaptic firing (Markram et al., 1997; Bi and Poo, 1998; Sjöström et al., 2001). When a pre-synaptic firing precedes a post-synaptic firing, the synapse is potentiated, whereas when a pre-synaptic firing follows a post-synaptic firing, the synapse is depressed. The STDP model is commonly expressed as follows:

(2) $$\Delta W(W,\Delta t)=\{\begin{array}{cc} A_{+}(W)\exp\left(-\frac{\left|\Delta t\right|}{\tau_{+}}\right) & \text{if }\Delta t<0\\ A_{-}(W)\exp\left(-\frac{\left|\Delta t\right|}{\tau_{-}}\right) & \text{if }\Delta t>0, \end{array}$$

where *W* is the synaptic weight and Δ*W* is the magnitude of the change in the synaptic weight *W*. The synaptic weight *W* is updated to *W* + Δ*W* immediately after whichever occurs later, the pre-synaptic firing (at *t*_pre_ in depression) or the post-synaptic firing (at *t*_post_ in potentiation). This is expressed as the temporal difference Δ*t* = *t*_post_ − *t*_pre_. The functions *A*_+_(*W*) and *A*_−_(*W*) determine the magnitudes Δ*W* of potentiation and depression, and the parameters τ_+_ and τ_−_ are the time constants of the magnitude Δ*W* decay. This study employed the multiplicative form of the STDP proposed by van Rossum et al. (2000), which is expressed as

(3) $$\begin{array}{c} A_{+}(W)=c_{+}+\nu_{p}W,\;A_{-}(W)=-c_{-}W+\nu_{p}W, \end{array}$$

where ν_*p*_ follows a normal distribution with zero mean and variance $\sigma_{p}^{2}$, i.e., $\nu_{p}\sim N\left(0,\sigma_{p}^{2}\right)$. A strong synapse is easily depressed and further potentiation of the strong synapse is prevented because the amount *A*_−_(*W*) of depression increases with synaptic weight *W*. Thus, the multiplicative STDP model is also referred to as soft-bounded STDP model (van Rossum et al., 2012). In this study, STDP was assumed to occur not between all possible pairs of pre- and post-synaptic firings but between the pair of a newly generated post-synaptic (pre-synaptic) firing and the latest pre-synaptic (post-synaptic) firing (Morrison et al., 2008; Watt and Desai, 2010). The parameter values were the same as those estimated in the original study by van Rossum et al. (2000): τ_+_ = τ_−_ = 20 ms, *c*_+_ = 1 pS, *c*_−_ = 0.003, and σ_*p*_ = 0.015.

### 2.3. Activity-dependent scaling

For comparison, a phenomenological model of activity-dependent scaling is introduced (van Rossum et al., 2000). This model monitors the activity of a post-synaptic neuron with a slow-varying sensor *a* as follows:

(4) $$\begin{array}{c} \tau_{a}\frac{da}{dt}=-a+\sum_{k}\delta (t-t_{k}), \end{array}$$

where δ(·) is the delta function, and *t*_*k*_ is the time of the *k*-th post-synaptic firing. At the state *a*, the weight *W* of a synapse connected to the neuron is regulated as follows:

(5) $$\frac{dW}{dt}=\beta W(a_{g}-a(t))+\gamma W\int_{0}^{t}(a_{g}-a(t^{\prime }))dt^{\prime },$$

where *a*_*g*_ (Hz) is the target post-synaptic firing rate. This formulation follows the classical proportional-integral controller. This study employed the same parameter values as those used in the original study, i.e., τ_*a*_ = 100 s, β = 4 × 10^−5^, and γ = 10^−7^ s^−1^, except that the target post-synaptic firing rate *a*_*g*_ was set to 5 Hz, which is more biologically realistic than the original value of 20 Hz (Mizuseki and Buzsáki, 2013; Buzsáki and Mizuseki, 2014). The features of these models are compared in Table 1.

**Table 1:** **Comparison between properties among models**.

**Model**	**no monitoring of neuronal activity**	**biological evidence**	**preventing low activity (Figure 2)**	**preventing high activity (Figure 2)**	**regulating with varying frequency (Figure 3A)**	**synaptic scaling (Figure 3B)**	**stability of synapses (Figure 4)**	**regulating under silenced activity (Figure 5)**	**competition between synapses (Figure 6)**	
+NO	+	+		+	+	−	+		+	With no additional factors
+LP	+	+			−	+	+			With a larger potentiation *c*_+_ = 1.5 pS
+IF	+	+	+	+	+	+	+	+	+	With intrinsic fluctuations^*^
+ADS			+	+	+	+	+	−	+	With activity−dependent scaling model^**^

“*+” denotes “having the property,” “−” denotes “having the property partially or with a problem,” and a blank denotes “not having the property.”*

* Yasumatsu et al. (2008)

** van Rossum et al. (2000)

### 2.4. Dynamics of neuron and synapses

The dynamics of the neuron were described by a leaky integrate-and-fire model (van Rossum et al., 2000; Izhikevich, 2004):

(6) $$\tau_{m}\frac{dv}{dt}=(v_{L}-v)+g_{E}(v_{E}-v)R+g_{I}(v_{I}-v)R,$$

where *v* is the membrane potential, τ_*m*_ = 20 ms is its time constant, and *v*_*L*_ = −60 mV, *v*_*E*_ = 0 mV, and *v*_*I*_ = −70 mV are the reversal potentials of leak, excitatory, and inhibitory currents, respectively. Hereafter, the subscripts *E* and *I* represent excitatory and inhibitory states, respectively; thus, *g*_*E*_ and *g*_*I*_ are the conductances of excitatory and inhibitory synaptic currents. *R* = 100 MΩ is the input resistance. When the membrane potential *v* reaches the threshold voltage *v*_*th*_ = −50 mV, it is reset to *v*_*r*_ = −60 mV immediately, and the neuron fires (i.e., generates an action potential). The number of post-synaptic firings per unit time is the post-synaptic firing rate *f*_post_. The dynamics of excitatory and inhibitory synaptic conductances are both described as follows:

(7) $$\frac{dg_{X}}{dt}=-\frac{g_{X}}{\tau_{X}}+\sum_{i}W_{i}\sum_{k}\delta \left(t-s_{i}^{\left(k\right)}\right)\text{ for }X\in \{E,I\},$$

where τ_*X*_ = 5 ms is the time constant of the conductance. The subscript *i* is the label of the synapse, *W*_*i*_ is the synaptic weight, and $s_{i}^{\left(k\right)}$ is the time of the *k*-th event at synapse *i*. The weights *W* of the excitatory synapses were plastic and followed the synaptic plasticity models described above. They are bounded at 0 to prevent a negative weight. The weights *W* of all inhibitory synapses were fixed at *W*_*I*_ = 4000 pS. All the parameter values were the same as those in the original study (van Rossum et al., 2000).

For numerical simulations, all the equations were solved by the forward Euler method with the time step *h* = 0.1 ms. A single post-synaptic neuron model was constructed that receives inputs from *N*_*f*_ = 100 excitatory and *N*_*f*_ ∕ 4 = 25 inhibitory synapses. The pre-synaptic firing rate was denoted by *f*_pre_ (Hz). The pre-synaptic firing rate per time step was *p* = *f*_pre_*h*. Excitatory pre-synaptic firings were assumed to be mutually correlated (van Rossum et al., 2000; Bair et al., 2001). This study generated a correlated input as a set of simultaneous excitatory pre-synaptic firings. A correlated group contained *N*_*c*_ excitatory synapses. An event within the correlated group followed a Poisson process of rate *p*_*c*_ per time step. For each event within the correlated group, *m* excitatory synapses were randomly chosen from the correlated group and were simultaneously activated. The rate *p*_*c*_ was set to *pN*_*c*_ ∕ *m* so that the pre-synaptic firing rate per time step was *p*. If the random variable *X*_*i*_ ∈ {0, 1} represents the occurrence of an event at the excitatory synapse *i*, the Pearson correlation coefficient between two excitatory synapses *i* and *j* in the correlated group is

(8) $$c=\frac{\text{cov}(X_{i},X_{j})}{\sigma_{X_{i}}\sigma_{X_{j}}}=\frac{\frac{pN_{c}}{m}\frac{{}_{N_{c}-2}{\text{C}}_{m-2}}{{}_{N_{c}}{\text{C}}_{m}}-p^{2}}{p(1-p)}=\frac{\frac{m-1}{N_{c}-1}-p}{1-p}≃\frac{m-1}{N_{c}-1}$$

as long as *p* ≪ 1. This study divided the excitatory synapses into four groups, i.e., *N*_*c*_ = 25. Thus,

(9) c≃0.04×(m−1).

In the case of uncorrelated inputs (i.e., *c* = 0), the excitatory synapses were activated by homogeneous Poisson processes at *f*_pre_ (Hz). In both cases, the inhibitory synapses followed homogeneous Poisson processes at *f*_pre_ (Hz). All the results were obtained from ten trials.

## 3. Results

### 3.1. Numerical simulations

#### 3.1.1. Stability of neuronal activity

**Figure 1:**
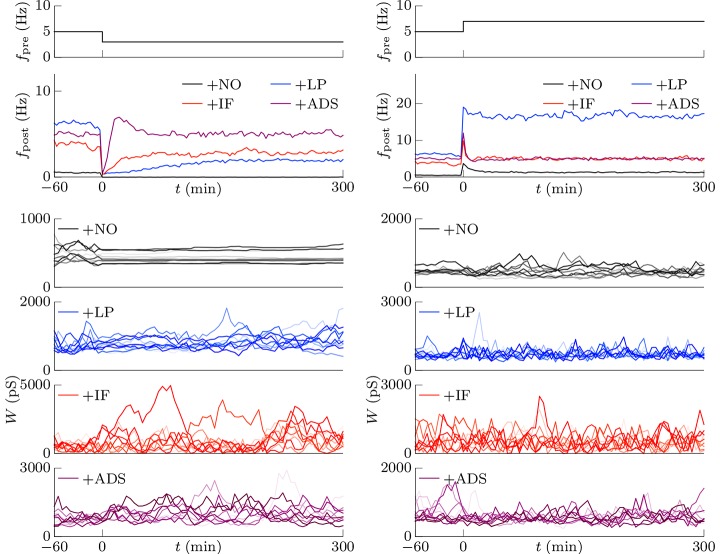
**Changes in post-synaptic firing rate *f*_post_ following a change in the pre-synaptic firing rate *f*_pre_**. Homeostatic plasticity is expected to restore the post-synaptic firing rate *f*_post_ to its previous (baseline) value. (Left panel) The pre-synaptic firing rate *f*_pre_ is decreased from 5 to 3 Hz. The different colored lines denote results of STDP models with no additional factors (+NO model, black), with a larger potentiation *c*+ = 1.5 pS (+LP model, blue), with intrinsic fluctuations (+IF model, red), and with activity-dependent scaling model (+ADS model, purple). The post-synaptic firing rates *f*_post_ of the +NO and +LP models decrease to approximately zero and never return to the previous baseline value. On the contrary, an excessively low post-synaptic firing rate *f*_post_ is prevented in the +IF and +ADS models. The trajectories of randomly chosen ten synapses are shown in the lower part. In the case of the +NO model, the synapses are almost unchanged owing to very low post-synaptic firing rate *f*_post_. In the other cases, some synapses become strong but others become weak; synapses are continuously shuffled even after the post-synaptic firing rate *f*_post_ converged to a steady state. (Right panel) The pre-synaptic firing rate *f*_pre_ is increased from 5 to 7 Hz. As is the case with the decreased pre-synaptic firing rate *f*_pre_, the +IF and +ADS models restore the post-synaptic firing rate *f*_post_ to the previous baseline value, whereas the +NO and +LP models do not.

This section examines the capacity of different STDP models (see Table 1) to maintain neuronal activity in response to a change in synaptic input (*f*_pre_). The excitatory synaptic weights *W* were initialized to a sufficiently large value, the synapses were activated with a pre-synaptic firing rate *f*_pre_ = 5 Hz and a correlation *c* = 0.04, and the post-synaptic neuron accepted the pre-synaptic firings. After a sufficiently long duration, the post-synaptic firing rate *f*_post_ in response to the pre-synaptic firings reached a steady state. The changes in post-synaptic firing rate *f*_post_ in response to a sudden decrease in the pre-synaptic firing rate *f*_pre_ are shown for each model in the left panel of Figure 1. The black line denotes the result obtained from the STDP model with no additional factors, which is referred to as the +NO model. The initial steady-state (baseline) post-synaptic firing rate *f*_post_ of this model in response to the pre-synaptic firing rate *f*_pre_ = 5 Hz was very low (< 0.1 Hz). At time *t* = 0, the pre-synaptic firing rate *f*_pre_ was decreased to 3 Hz to simulate a condition such as sensory deprivation (Keck et al., 2013). After this decrease, the post-synaptic firing rate *f*_post_ decreased to approximately zero and never returned to its previous value. To prevent this excessively low post-synaptic firing rate *f*_post_, the amplitude *c*_+_ of potentiation was increased to 1.5 pS in a model referred to hereafter as +LP model, (blue lines in Figure 1). A larger potentiation *c*_+_ = 1.5 pS induced larger synaptic weights *W* and a higher baseline post-synaptic firing rate *f*_post_ in response to the pre-synaptic firing rate *f*_pre_ = 5 Hz. After decreasing the pre-synaptic firing rate *f*_pre_, the post-synaptic firing rate *f*_post_ temporally fell to zero and quickly started to increase. However, it did not return to baseline. Both models cannot prevent excessively low neuronal activity. In contrast, the model incorporating intrinsic fluctuations (red line in Figure 1), hereafter denoted as the +IF model, prevented this drastic decrease in the post-synaptic firing rate *f*_post_ and restored it to baseline, thereby indicating homeostatic plasticity. For comparison, the synaptic plasticity model incorporating activity-dependent scaling and STDP (the +ADS model, purple line in Figure 1) also showed a rapid return to the baseline post-synaptic firing rate *f*_post_ after an overshoot. Ten synapses were randomly chosen and their trajectories are shown in the lower part of Figure 1. In the case of the +NO model, the synapses were almost unchanged owing to very low post-synaptic firing rate *f*_post_. In the other cases, some synapses became strong but others became weak; synapses were continuously shuffled even after the post-synaptic firing rate *f*_post_ converged to a steady state.

The pre-synaptic firing rate *f*_pre_ was then increased from 5 to 7 Hz to simulate increased neuronal activity (right panel of Figure 1). In all four models, the post-synaptic firing rate *f*_post_ decreased after an initial increase. However, similar to the case of the simulation of decreased pre-synaptic firing rate *f*_pre_, the +IF and +ADS models showed rapid restoration of post-synaptic firing rate *f*_post_ to the baseline value, whereas the +NO and +LP models did not. Thus, the +IF and +ADS models prevented excessive changes in neuronal activity and thereby demonstrated homeostatic plasticity. The trajectories of synapses are also shown in the lower part. In cases with the +IF and +ADS models, synapses were depressed on average. More detailed analyses are provided in the following sections.

**Figure 2:**
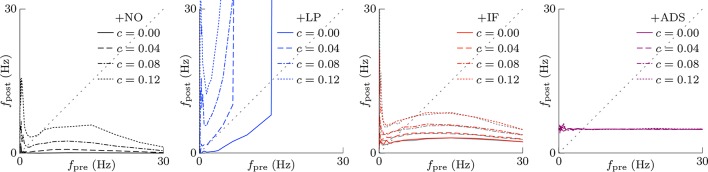
**The post-synaptic firing rates *f*_post_ in response to varying the pre-synaptic firing rate *f*_pre_ and the correlation *c***. The axis of abscissas represents to the pre-synaptic firing rate *f*_pre_ and the line type refers to the correlation *c*. The +NO, +LP, +IF, and +ADS models are all STDP models but with no additional factors, a larger potentiation *c*+ = 1.5 pS, intrinsic fluctuations, or activity-dependent scaling model. At a high pre-synaptic firing rate *f*_pre_ and a low correlation *c*, the +NO and +LP models cannot prevent a near-zero post-synaptic firing rate *f*_post_. The +LP model also cannot prevent an excessively high post-synaptic firing rate *f*_post_. On the other hand, the +IF and +ADS models maintain the post-synaptic firing rate *f*_post_ within a functional range, indicating that intrinsic fluctuations and activity-dependent scaling model achieve homeostatic plasticity. The gray lines in the third panel denote the results obtained from the +IF model without the noise term ν_*p*_, which were almost the same as those obtained from the +IF model with the noise term ν_*p*_ denoted by the red lines.

In subsequent examinations, the pre-synaptic firing rate *f*_pre_ and the correlation *c* of the pre-synaptic firings were widely varied. Figure 2 shows the relationship between pre-synaptic firing rate *f*_pre_ and resulting post-synaptic firing rates *f*_post_ at steady state. In the +NO model, the post-synaptic firing rate *f*_post_ reached a value of ≥1 Hz over a range of pre-synaptic firing rate *f*_pre_ = 0.01–20 Hz only with a high correlation *c*≥0.08. With a low correlation *c* < 0.08, the post-synaptic firing rate *f*_post_ decreased to approximately zero (*f*_post_ < 0.1 Hz), regardless of the pre-synaptic firing rate *f*_pre_. Thus, the soft-bounded STDP model prevented excessively strong synaptic weights *W* but simultaneously leaded to a post-synaptic firing rate *f*_post_ of approximately zero because of positive feedback. The +LP model was expected to prevent this excessively low post-synaptic firing rate *f*_post_ because of a larger potentiation (*c*_+_ = 1.5 pS). However, again the post-synaptic firing rate *f*_post_ fell to approximately zero at a pre-synaptic firing rate *f*_pre_ of < 1 Hz. Moreover, the post-synaptic firing rate *f*_post_ rose to >100 Hz at a high pre-synaptic firing rate *f*_pre_ > 20 Hz or a high correlation *c* > 0.04. This result indicates that increasing the amplitude *c*_+_ of potentiation is not enough for preventing almost silent activity and leads to a runaway excitatory as a side effect. As mentioned in the previous studies, the ranges of parameter values with which soft-bounded STDP models provide stability of neuronal activity are strictly limited (Morrison et al., 2007; Gilson and Fukai, 2011; Yger and Gilson, 2015). On the other hand, the +IF model maintained a post-synaptic firing rate *f*_post_ within 3–10 Hz over a broad range of pre-synaptic firing rate *f*_pre_ (0.01–30 Hz) and correlation *c* (0–0.12) values because the intrinsic fluctuations potentiated synapses with near zero weights (as mentioned in Section 2.1). The post-synaptic firing rate *f*_post_ varied little with pre-synaptic firing rate *f*_pre_ but nonetheless increases with correlation *c* (Figure 2, third panel, red lines). The +IF model has doubly noise terms; intrinsic fluctuations and the noise term $\nu_{p}\sim N\left(0,\sigma_{p}^{2}\right)$ in potentiation and depression of soft-bounded STDP model. The +IF model without the noise term ν_*p*_ (σ_*p*_ = 0) was also examined (Figure 2, third panel, gray lines). The results were almost the same as those obtained from the +IF model with the noise term ν_*p*_. For comparison, the +ADS model that incorporates both the activity-dependent scaling model and the STDP model, was examined. In this case, regardless of pre-synaptic firing rate *f*_pre_ and correlation *c*, the resultant post-synaptic firing rate *f*_post_ was almost similar to the target post-synaptic firing rate *a*_*g*_ = 5 Hz.

This section concludes that intrinsic fluctuations maintain neuronal activity within limits in response to changing conditions (pre-synaptic firing rate and correlation) and can mediate homeostatic plasticity (see Table 1).

#### 3.1.2. Scaling of synaptic weights

**Figure 3:**
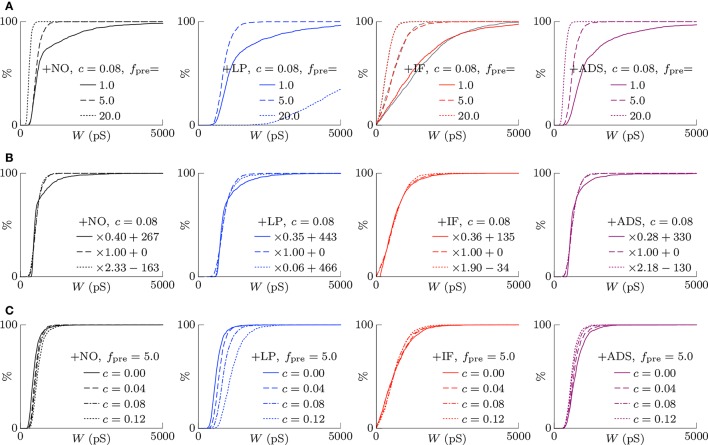
**The cumulative distributions of the excitatory synaptic weights *W* at steady states with varying pre-synaptic firing rate *f*_pre_ or correlation *c***. **(A)** The pre-synaptic firing rate *f*_pre_ is varied. **(B)** The pre-synaptic firing rate *f*_pre_ is varied and the synaptic weights *W* are scaled appropriately. **(C)** The correlation *c* is varied. Colors as in Figures 1, 2.

According to the hypothesis of synaptic scaling (Turrigiano et al., 1998; Ibata et al., 2008; Keck et al., 2013), neuronal activity is adjusted by scaling the excitatory synaptic weights multiplicatively. To confirm this hypothesis, Figure 3 summarizes the cumulative distributions of synaptic weights *W* at steady states for the four models. First, the effects of changing pre-synaptic firing rate *f*_pre_ are shown in Figure 3A. The +NO, +IF, and +ADS models all yielded lower synaptic weights *W* with progressive increases in pre-synaptic firing rate *f*_pre_; these results are consistent with the synaptic scaling observed in previous biological experiments (Turrigiano et al., 1998; Ibata et al., 2008; Keck et al., 2013). Contrary to the previous theoretical studies (van Rossum et al., 2000; Gilson and Fukai, 2011), the +NO model (the STDP model with no additional factors) also achieved synaptic scaling. A pre-synaptic firing always has a chance to induce post-synaptic firing and potentiation. With decreasing pre-synaptic firing rate *f*_pre_, the chance that a post-synaptic firing immediately precedes a pre-synaptic firing decreases (as they become sparse); thus, the probability of depression decreases and the synapses are potentiated more easily (see Section 3.2 for details). However, this potentiation is not sufficient for the post-synaptic neuron to elicit action potentials above a certain rate, as shown in Section 3.1.1. In the +LP model with pre-synaptic firing rate *f*_pre_ = 20 Hz, the synaptic weights *W* become exceptionally high. According to Figure 2, the post-synaptic firing rate *f*_post_ diverged from baseline, representing runaway excitation. Therefore, all of these models can adjust synaptic weight, but only the +IF and the +ADS models can adjust it sufficiently to prevent runaway excitation and silence (see Table 1). The +IF model without the noise term ν_*p*_ (σ_*p*_ = 0) was also examined (Figure 3A, third panel, gray lines). The results were also almost the same as those obtained from the +IF model with the noise term ν_*p*_.

The synaptic weights *W* were scaled so that their first and third quartiles were constant with varying pre-synaptic firing rate *f*_pre_. The results are summarized in Figure 3B. The shapes of the cumulative distributions of the synaptic weights *W* were similar in most cases; however, the +NO model with pre-synaptic firing rate *f*_pre_ = 1 Hz yielded a different shape. This indicates that STDP, which is incorporated into all models examined, achieves synaptic scaling by itself.

Next, the correlation *c* is varied. It is to be noted that the original biological experiments (Turrigiano et al., 1998; Ibata et al., 2008; Keck et al., 2013) did not refer to synaptic scaling induced by varying the correlation *c* because varying correlation *c* is difficult in biological systems (but not *in silico*). The results are summarized in Figure 3C. The +NO and +LP models show increased synaptic weights *W* with higher correlation *c*, indicating that these models do not provide negative feedback against increasing correlation *c*. With increase in the correlation *c*, the +IF model exhibits a decreased number of strong synapses but a greater number of weak synapses. The +ADS model shows depression of all synapses with increasing correlation *c*. Because synaptic scaling induced by varying the correlation *c* has not been examined *in vivo*, this examination cannot draw biological conclusions.

#### 3.1.3. Stability of strong synapses

**Figure 4:**
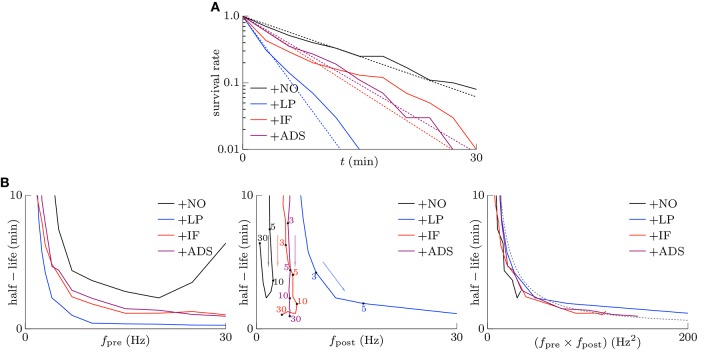
**Stability of strong excitatory synapses**. Synapses whose weights are stronger than the 90th percentile of all the synaptic weights of each respective trial at *t* = 0 are treated as strong. **(A)** The solid lines denote the survival rate of the strong synapses in response to the pre-synaptic firing with the rate of *f*_pre_ = 5 Hz and the correlation of *c* = 0.08. The dotted lines denote the half-lives estimated by maximum likelihood; 7.5, 1.9, 4.0, and 4.4 min for +NO, +LP, +IF and +ADS models respectively. **(B)** The estimated half-lives with varying pre-synaptic firing rate *f*_pre_. The axes of abscissas represent the pre-synaptic firing rate *f*_pre_ (left), the post-synaptic firing rate *f*_post_ (middle), and the product *f*_pre_ *f*_post_ of them (right). The numerals in the middle panel indicate the corresponding pre-synaptic firing rates *f*_pre_ (Hz).

Memory is considered to be stored as synaptic connections and thus strong synapses should stay strong (Fusi et al., 2005; Fusi and Abbott, 2007; Morrison et al., 2007; Gilson and Fukai, 2011). Intrinsic fluctuations however have a risk disturbing retention of memory by depressing strong synapses since they are random fluctuations. However, not only intrinsic fluctuations but also the soft-bounded STDP model shuffles synapses (see the lower part of Figure 1). Effects of intrinsic fluctuations on stability of strong synapses were examined. The pre-synaptic firing rate *f*_pre_ and the correlation *c* were set to 5 Hz and *c* = 0.08, and the post-synaptic firing rates *f*_post_ reached a steady state. Synapses whose weights are stronger than the 90th percentile of all the synaptic weights of each respective trial at *t* = 0 were treated as strong. The survival rates, i.e., the rates of the synapses remaining strong from *t* = 0, are depicted as the solid lines in Figure 4A. The following half-lives were estimated by maximum likelihood; 7.5, 1.9, 4.0, and 4.4 min for the +NO, +LP, +IF, and +ADS models, respectively (see the dotted lines in Figure 4A). In this case, the post-synaptic firing rates *f*_post_ were 2.02, 16.37, 5.23, and 4.97 Hz for the +NO, +LP, +IF, and +ADS models, respectively (see Figure 2). The estimated half-lives with varying pre-synaptic firing rate *f*_pre_ are shown in the left panel of Figure 4B; the half-lives obtained from the +IF model were almost equal to those from the +ADS model, shorter than those from the +NO model, and longer than those from the +LP model. The increasing order of the half-lives was similar to the decreasing order of post-synaptic firing rates *f*_post_ (see also Figure 2). Only with a small pre-synaptic firing rate *f*_pre_, the +IF model resulted in a shorter half-life despite the lower post-synaptic firing rate *f*_post_ when compared with the +ADS model. The middle panel of Figure 4B shows the same results except that the axis of abscissas represents the post-synaptic firing rate *f*_post_ instead of the pre-synaptic firing rate *f*_pre_. Owing to homeostatic function, the post-synaptic firing rates *f*_post_ were varied a little with the +IF and +ADS models. No obvious relationship between the half-life and the post-synaptic firing rate *f*_post_ was found. The right panel has the product *f*_pre_ × *f*_post_ of the pre- and post-synaptic firing rates on the axis of abscissas. The dotted gray line represents 130∕(*f*_pre_ × *f*_post_) (min · Hz^−2^). The half-life was roughly inversely proportional to the product *f*_pre_ × *f*_post_ of the pre- and post-synaptic firing rates; the product *f*_pre_ × *f*_post_ is also roughly proportional to the occurrence probability of STDP (van Rossum et al., 2000; Gilson and Fukai, 2011) (see also Section 3.2.1). These results suggest that the half-life (i.e., the stability) of the strong synapse is mainly related to the neuronal activity and the resulting occurrence probability of STDP. Intrinsic fluctuations have a slightly negative influence on the stability of strong synapses but it is almost negligible as long as the neuronal activity (i.e., the occurrence probability of STDP) is not too small.

#### 3.1.4. Scaling after silenced neuronal activity

**Figure 5:**
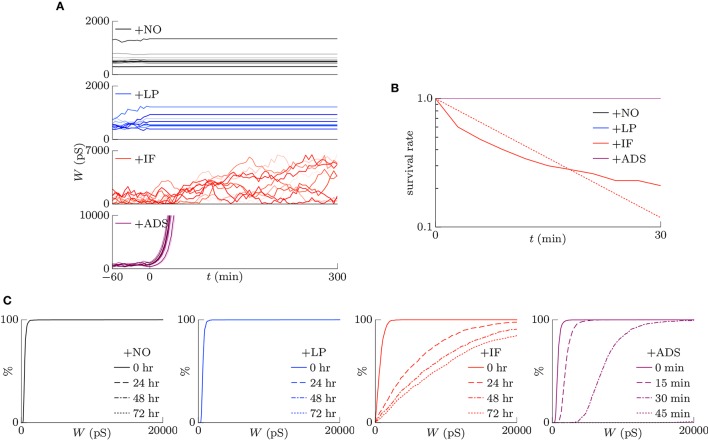
**Excitatory synaptic weights *W* in transient states after neuronal activity is silenced**. The correlation *c* is 0, and the pre-synaptic firing rate *f*_pre_ is decreased from 5 to 0 Hz at *t* = 0. **(A)** The trajectories of randomly chosen ten synapses. The +NO and +LP models cannot regulate the synaptic weights after the pre-synaptic firing rate *f*_pre_ is silenced; the synaptic weights are unchanged. The +IF model shuffles synaptic weights continuously and slowly potentiates synaptic weights on average. The +ADS model potentiates synaptic weights drastically; the trajectories are clipped for visibility. **(B)** The survival rates of the synapses remaining stronger than the 90th percentile. The +ADS model keeps the order of weight since it potentiates synapses multiplicatively. The survival rates obtained from the +NO, +LP, and +ADS models are 1.0. The +IF model has a probability for depressing the strong synapses; the half-life is 9.7 min. Note that the half-life is obtained in a transient state differently from the half-life in Figure 4, which is obtained in steady state. **(C)** The cumulative distributions of the excitatory synaptic weights *W*. The +IF model potentiates the synaptic weights *W* over several days. The +ADS model potentiates the synaptic weights *W*, but the post-synaptic firing rate *f*_post_ never returns to the target post-synaptic firing rate ag (Hz) because the pre-synaptic firing rate *f*_pre_ is zero. Consequently, the synaptic weights *W* become infinite.

Homeostatic plasticity has been also found during prolonged bath application of the voltage-gated sodium channel antagonist tetrodotoxin (TTX), which silences neuronal activity (Turrigiano et al., 1998; Ibata et al., 2008). This section highlights the examination of the cumulative distribution of excitatory synaptic weights *W* in transient states after the pre-synaptic firing rate *f*_pre_ is reduced to zero. The correlation *c* was set to 0, and the pre-synaptic firing rate *f*_pre_ was decreased from 5 to 0 Hz at *t* = 0. The results are shown in Figure 5. Figure 5A shows the trajectories of randomly chosen ten synapses. The +NO and +LP models did not regulate the synaptic weights after the pre-synaptic firing rate *f*_pre_ was silenced; the synaptic weights were unchanged. The +IF model shuffled synaptic weights continuously and slowly potentiated synaptic weights on average. The +ADS model potentiated synaptic weights drastically; the trajectories obtained from the +ADS model were clipped for visibility. Figure 5B shows the survival rates of the synapses remaining stronger than the 90th percentile. The +ADS model kept the order of weight since it potentiated synapses multiplicatively. The survival rates obtained from the +NO, +LP, and +ADS models are 1.0. Since intrinsic fluctuations induce change with zero expected value, the +IF model had probabilities for potentiating the weak synapses and depressing the strong synapses; the half-life is 9.7 min. As mentioned in Section 3.1.3, their negative influence on the stability of the strong synapses is no longer negligible. Furthermore, the half-life was obtained in a transient state differently from the half-life in Figure 4, which was obtained in steady state. The synaptic weights are considered to be less stable than those in steady state. Figure 5C shows the cumulative distributions of the excitatory synaptic weights *W*. The +IF model potentiated the synaptic weights *W* over several days. The +ADS model potentiated the synaptic weights *W*, but the post-synaptic firing rate *f*_post_ never returned to the target post-synaptic firing rate *a*_*g*_ (Hz) because the pre-synaptic firing rate *f*_pre_ was zero. Consequently, the synaptic weights *W* became infinite. This result is not consistent with that of the previous biological experiments (Turrigiano et al., 1998; Turrigiano, 1999, 2008; Burrone et al., 2002; Ibata et al., 2008; Keck et al., 2013; Félix-Oliveira et al., 2014). Hence, based on these results, it can be concluded that the +IF and +ADS models achieve synaptic scaling even after neuronal activity is silenced, but the +ADS model exhibits strange behavior (yields saturated synaptic weights *W*) under this condition (see Table 1 for comparison). A theoretical analysis is provided in **Figure 7A**.

#### 3.1.5. Competition

**Figure 6:**
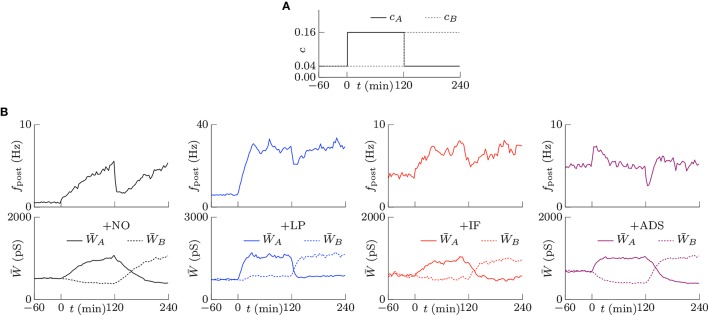
**The competition between the correlated groups (A,B) of excitatory synapses**. The time courses of the correlations *cA* and *cB*. **(A)** The time courses of the post-synaptic firing rates *f*_post_. **(B)** The time courses of the average synaptic weights $\bar{W}$ of the groups *A* and *B*. The +NO, +IF, and +ADS models all exhibit the competition, while the +LP model do not.

Co-occurring excitatory pre-synaptic firings have a greater chance to induce post-synaptic firing because of summation, thereby potentiating synapses. In contrast to this type of cooperation, synaptic plasticity is also competitive (Miller, 1996; Song et al., 2000; van Rossum et al., 2000; Gütig et al., 2003; Morrison et al., 2007; Gilson and Fukai, 2011); when a group of excitatory synapses has an effect, another group is depressed. Competition between synapses contributes to selectivity of neurons for different input patterns. The traditional STDP models exhibit strong competition often with destabilization of synaptic weights, the soft-bounded STDP models exhibit weak competition, and the activity-dependent scaling model promotes competition. Similar to the activity-dependent scaling model, intrinsic fluctuations are expected to contribute to competition. To examine the competition achieved by synaptic plasticity, two groups *A* and *B* of excitatory synapses were chosen from the four groups. Groups *A* and *B* had the same pre-synaptic firing rate *f*_pre_ = 5.0 Hz and had the same or different correlations *c*_*A*_ and *c*_*B*_. First, the correlations were set to *c*_*A*_ = *c*_*B*_ = 0.04. After a sufficiently long duration, the correlation *c*_*B*_ was increased to *c*_*B*_ = 0.16 at *t* = 0, as shown in Figure 6A. The resulting changes in the post-synaptic firing rate *f*_post_ and synaptic weights *W* are summarized in Figure 6B. In all cases, the post-synaptic firing rate *f*_post_ increased to a larger value at time *t* = 0 and the average weight ${\bar{W}}_{A}$ of group *A* synapses started increasing. The response of the average weight ${\bar{W}}_{B}$ of group *B* synapses depended on the plasticity model. In the +NO model, the average weight ${\bar{W}}_{B}$ of group *B* was depressed. In other words, the model resulted in competition between synapses. The +IF and +ADS models also exhibited competition. On the other hand, the average weight ${\bar{W}}_{B}$ was almost unchanged in the +LP model. Thus, whether soft-bounded STDP model exhibits competition depends on parameter values (e.g., the amplitude *c*_+_ of the potentiation). A theoretical analysis is shown in Section 3.2.3.

### 3.2. Theoretical analyses

#### 3.2.1. Under uncorrelated activity

In this section, a procedure to obtain a synaptic weight distribution *P*(*W*) theoretically is explained, and the mechanisms of homeostatic plasticity and competition are analyzed. The synaptic modifications Δ*W* yielded by different synaptic plasticity models can be treated as stochastic differential equations as long as Δ*W* are sufficiently small. A synaptic weight distribution *P*(*W*) is obtained using the Fokker-Planck equation (Øksendal, 2003) with the moments M_*n*_ of the synaptic modifications Δ*W*, where the moments M_*n*_ are derived as

(10) $$M_{n}(W)=\int P(\Delta W)\text{E}[\Delta W{\left(W\right)}^{n}]d\Delta W.$$

First, for simplicity, post-synaptic firing is assumed to be unrelated to pre-synaptic firings and to follow a single Poisson process of *f*_post_ (Hz), which is obtained from the numerical simulations in Section 3.1. This assumption is reasonable when a post-synaptic neuron accepts inputs from numerous synapses or its membrane potential is noisy. In addition, STDP is assumed to occur between all possible pairs of pre- and post-synaptic firings (all-to-all STDP model Morrison et al., 2008; Watt and Desai, 2010) also for simplicity. This approximation is reasonable as long as pre- and post-synaptic neuron firing rates are not excessively high (Gilson and Fukai, 2011). The *n*-th moments $M_{n}^{STDP}\left(W\right)$ of the synaptic modifications yielded by the STDP model are derived as follows (van Rossum et al., 2000; Yasumatsu et al., 2008; Kasai et al., 2010; Gilson and Fukai, 2011; Statman et al., 2014):

(11) $$\begin{array}{l} M_{n}^{\text{STDP}}(W)=\int_{-\text{​}\infty }^{\infty }P(\Delta W)\text{E}[\Delta W{\left(W\right)}^{n}]d\Delta W\\ \;=f_{\text{post}}\int_{-\text{​}\infty }^{0}\text{E}[\Delta W{\left(W,\Delta t\right)}^{n}]P(\Delta t)d\Delta t\\ \;+f_{\text{pre}}\;\int_{0}^{\infty }\text{E}[\Delta W{\left(W,\Delta t\right)}^{n}]P(\Delta t)d\Delta t\\ \;=f_{\text{pre}}f_{\text{post}}\int_{-\text{​}\infty }^{0}\text{E}[A_{+}\text{​}{\left(W\right)}^{n}]\exp\left(-n\frac{\left|\Delta t\right|}{\tau_{+}}\right)d\Delta t\\ \;+f_{\text{pre}}f_{\text{post}}\int_{0}^{\infty }\text{E}[A_{-}\text{​}{\left(W\right)}^{n}]\exp\left(-n\frac{\left|\Delta t\right|}{\tau_{-}}\right)d\Delta t\\ \;=\frac{1}{n}f_{\text{pre}}f_{\text{post}}\left(\tau_{+}\text{E}[A_{+}{\left(W\right)}^{n}]+\tau_{-}\text{E}[A_{-}{\left(W\right)}^{n}]\right), \end{array}$$

where *P*(Δ*W*) and *P*(Δ*t*) are the occurrence probabilities of the potentiation (or depression) with the amount of Δ*W* and with the temporal difference Δ*t*. *P*(Δ*t*) is equal to the pre-synaptic firing rate *f*_pre_ when Δ*t* > 0 and to the post-synaptic firing rate *f*_post_ when Δ*t* < 0 because of the all-to-all STDP model. Because this study employs the multiplicative STDP model, described in Equation (3), the moments M_*n*_ are as follows:

(12) $$\begin{array}{l} M_{1}^{\text{STDP}}(W)=f_{\text{pre}}f_{\text{post}}\left(\tau_{+}c_{+}-\tau_{-}c_{-}W\right),\\ M_{2}^{\text{STDP}}(W)=\frac{1}{2}f_{\text{pre}}f_{\text{post}}(\tau_{+}(c_{+}^{2}+\sigma_{p}^{2}W^{2})\\ \;+\tau_{-}(c_{-}^{2}W^{2}+\sigma_{p}^{2}W^{2})). \end{array}$$

In addition to STDP, the +IF and +ADS models take into account synaptic modifications yielded by intrinsic fluctuations and the activity-dependent scaling model, respectively. The moments $M_{n}^{H}$ of their synaptic modifications are called as the homeostatic terms in this study and expressed as follows (van Rossum et al., 2000; Yasumatsu et al., 2008; Kasai et al., 2010):

(13) $$\begin{array}{l} M_{1}^{\text{H}}(W)=\{\begin{array}{ll} \beta W(a_{g}-a(t)) & \text{for the}+\text{ADS​ model},\\ \;+\gamma W\int_{0}^{t}(a_{g}-a(t^{\prime }))dt^{\prime } & \\ 0 & \text{for other models}. \end{array}\\ M_{2}^{\text{H}}(W)=\{\begin{array}{ll} {\left(SW+s\right)}^{2} & \text{for the}+\text{IF model},\\ 0 & \text{for other models}. \end{array} \end{array}$$

Because of the assumption that the post-synaptic firing follows a single Poisson process of *f*_post_, *a*(*t*) is clamped to *a*_*g*_ and the activity-dependent scaling model does not work. Thus, the moments M_*n*_ of the synaptic modifications are as follows:

(14) $$\begin{array}{l} M_{1}(W)=M_{1}^{\text{STDP}}(W)+M_{1}^{\text{H}}(W),\\ M_{2}(W)=M_{2}^{\text{STDP}}(W)+M_{2}^{\text{H}}(W). \end{array}$$

The Fokker-Planck equation describes the changes in the synaptic weight distribution *P*(*W, t*) as follows:

(15) $$\frac{\partial }{\partial t}P(W,t)=-\frac{\partial }{\partial W}M_{1}(W)P(W,t)+\frac{1}{2}\frac{\partial^{2}}{\partial W^{2}}M_{2}(W)P(W,t).$$

**Figure 7:**
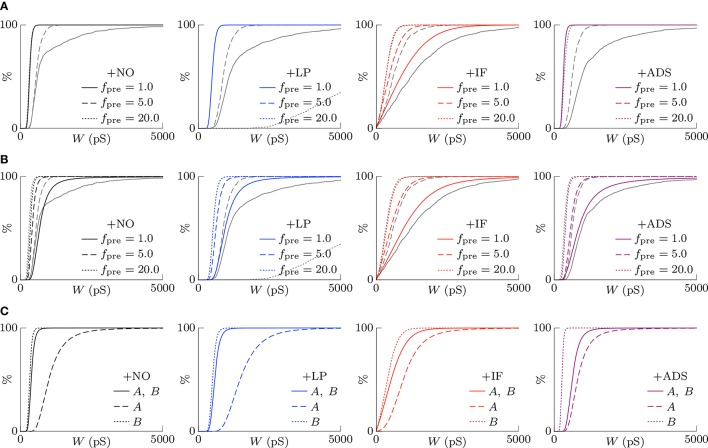
**The theoretically-obtained cumulative distributions of excitatory synaptic weights *W***. The black and colored lines denote the theoretical results and the gray lines denote the corresponding numerical results shown in Figure 3A. **(A)** Post-synaptic firing is assumed to be unrelated to pre-synaptic firings and to follow a single Poisson process of *f*_post_ (Hz) (see Equation 14). Synaptic scaling is observed only in the case of the +IF model. The theoretical results and the numerical results are almost different except for the case of the +IF model. **(B)** The dependency of post-synaptic firing on pre-synaptic firings is taken into account (see Equation 17). All the models achieve synaptic scaling. By taking into account the dependency terms $M_{n}^{Dep}\left(W\right)$, the theoretical results closely resemble the numerical results except for the +LP model with a high pre-synaptic firing rate *f*_pre_ = 20.0. **(C)** The cumulative distributions of excitatory synaptic weights *W* under the competitive situation. The solid lines denote the synaptic weight distribution *P*(*W*) when correlated excitatory synapse groups *A* and *B* had the same correlation *c*_*A*_ = *c*_*B*_ = 0.04. The dashed lines denote the synaptic weight distribution *P*_*A*_(*W*) of correlated group *A* after its correlation *c*_*A*_ is increased to 0.16. The synapses are potentiated. The dotted lines denote the synaptic weight distribution *P*_*B*_(*W*) of correlated group *B* after *c*_*A*_ is increased to 0.16. When the synapses in the correlated group *B* are depressed, synaptic scaling is achieved.

After a sufficiently long duration, the synaptic weight distribution *P*(*W, t*) converges to a steady state, which is denoted by *P*(*W*). Because the left side is equal to zero, the steady state synaptic weight distribution *P*(*W*) is

(16) $$P(W)=\frac{C}{M_{2}(W)}\exp\left(\int_{0}^{W}\frac{2M_{1}(W^{\prime })}{M_{2}(W^{\prime })}dW^{\prime }\right)$$

where *C* is a normalization factor. Figure 7A shows the theoretically-obtained synaptic weight distributions *P*(*W*). The colored lines denote the theoretical results and the gray lines denote the corresponding numerical results shown in Figure 3A. They are almost different except for the case of the +IF model. In the absence of intrinsic fluctuations, both the first moment M_1_(*W*) and the second moment M_2_(*W*) are directly proportional to the product of the pre-synaptic firing rate *f*_pre_ and the post-synaptic firing rate *f*_post_. They cancel out in Equation (16), and the synaptic weight distribution *P*(*W*) is independent of neuronal activity. In the presence of intrinsic fluctuations, the second moment M_2_(*W*) is the sum of the activity-dependent term $M_{2}^{STDP}\left(W\right)$ and the activity-independent term $M_{2}^{H}\left(W\right)$; thus, the synaptic weight distribution *P*(*W*) depends on the product of the pre-synaptic firing rate *f*_pre_ and the post-synaptic firing rate *f*_post_. With increasing pre-synaptic firing rate *f*_pre_, the activity-independent term $M_{2}^{H}\left(W\right)$ is relatively negligible and the synaptic weight distribution *P*(*W*) resembles that in the absence of intrinsic fluctuations. With decreasing pre-synaptic firing rate *f*_pre_, the activity-independent term $M_{2}^{H}\left(W\right)$ is dominant and the synaptic weights are widely distributed because of the relatively large diffusion term M_2_(*W*). This is the mechanism by which the +IF model regulates synaptic weights *W* against a change in neuronal activity.

In the case of silenced neuronal activity (*f*_pre_ = 0), the activity-dependent terms $M_{n}^{STDP}\left(W\right)$ are zero. Thus, the synaptic weight distributions *P*(*W*) obtained from the +NO and +LP models are unchanged. Owing to the activity-independent term $M_{2}^{H}\left(W\right)$, the +IF and +ADS models mediate synaptic weight. The steady-state synaptic weight distribution *P*(*W*) obtained from the +IF model is a power-law distribution $\frac{Ss}{{\left(SW+s\right)}^{2}}$ with the exponent of 2; it has no well-defined mean weight. The steady-state synaptic weight distribution *P*(*W*) obtained from the +ADS model cannot be well-defined; activity-dependent scaling model keeps on potentiating synaptic weights unboundedly. Both the +IF and +ADS models lose stability of synaptic weight distribution in the case of silenced neuronal activity, but the +IF model needs much longer duration than the +ADS model according to the numerical simulations (see Figure 5).

#### 3.2.2. Under correlated activity

The pre-synaptic firings have a strong impact on post-synaptic firing. Events at strong synapses or correlated synapses have greater chances to induce post-synaptic firing and potentiation. Next, the dependency of post-synaptic firing on pre-synaptic firings is taken into account. The excitatory synapses are divided into the correlated groups labeled *l*, each containing *N*_*c, l*_ excitatory synapses. A set of *m*_*l*_ excitatory synapses in a correlated group *l* is simultaneously stimulated. A pre-synaptic firing with a weight *W* in a correlated group *l* has a chance $P_{l}^{fire}\left(W\right)$ to induce post-synaptic firing. Each group *l* shares the post-synaptic firing rate *f*_post_, but has its own synaptic weight distribution *P*_*l*_(*W*). Equation (14) can be rewritten as

(17) $$\begin{array}{l} M_{1,l}(W)=M_{1,l}^{\text{STDP}}(W)+M_{1,l}^{\text{H}}(W)+M_{1,l}^{\text{Dep}}(W),\\ M_{2,l}(W)=M_{2,l}^{\text{STDP}}(W)+M_{2,l}^{\text{H}}(W)+M_{2,l}^{\text{Dep}}(W), \end{array}$$

where the terms $M_{n,l}^{Dep}\left(W\right)$ are the dependency terms. Under the simplified condition that the post-synaptic neuron fires immediately after the pre-synaptic firing, i.e., Δ*t* < 0 and |Δ*t*| ≪ 1, the dependency terms $M_{n,l}^{Dep}\left(W\right)$ are expressed as

(18) $$\begin{array}{l} M_{1,l}^{\text{Dep}}(W)=f_{\text{pre}}P_{l}^{fire}(W)\text{E}[A_{+}(W)]=f_{\text{pre}}P_{l}^{fire}(W)c_{+},\\ M_{2,l}^{\text{Dep}}(W)=f_{\text{pre}}P_{l}^{fire}(W)\text{E}[A_{+}{\left(W\right)}^{2}]=f_{\text{pre}}P_{l}^{fire}(W)\\ \;(c_{+}^{2}+\sigma_{p}^{2}W^{2}). \end{array}$$

The firing chance $P_{l}^{fire}\left(W\right)$ depends on the probability distribution *P*(*v*) over time of the membrane potential *v*. A detailed derivation of the firing chance $P_{l}^{fire}\left(W\right)$ is shown in Appendix. Unlike the STDP terms $M_{n,l}^{STDP}\left(W\right)$, the dependency terms $M_{n,l}^{Dep}\left(W\right)$ are proportional to the pre-synaptic firing rate *f*_pre_, but not to the post-synaptic firing rate *f*_post_. Thus, the ratio between the first moment M_1_(*W*) and the second moment M_2_(*W*) depends on the post-synaptic firing rate *f*_post_. The post-synaptic firing rate *f*_post_ is assumed to be

(19) $$f_{\text{post}}=f_{\text{pre}}\sum_{l}\frac{N_{c,l}}{m_{l}}\text{E}[P_{l}^{fire}(W)].$$

This value is used for the STDP terms $M_{n,l}^{STDP}\left(W\right)$. Figure 7B shows the theoretical synaptic weight distributions *P*(*W*) taking into account the dependency terms $M_{n}^{Dep}\left(W\right)$. The colored lines denote the theoretical results and the gray lines denote the corresponding numerical results shown in Figure 3A. By taking into account the dependency terms $M_{n}^{Dep}\left(W\right)$, the theoretical results closely resemble the numerical results.

In the cases of the +NO and +LP models, the synapses are potentiated with a low pre-synaptic firing rate *f*_pre_ and conversely the synapses are depressed with a high pre-synaptic firing rate *f*_pre_. The relationships between the STDP terms $M_{n,l}^{STDP}\left(W\right)$ and the dependency terms $M_{n,l}^{Dep}\left(W\right)$ of the correlated group *l* are

(20) $$\begin{array}{l} \frac{M_{n,l}^{\text{STDP}}(W)}{M_{n,l}^{\text{Dep}}(W)}=\frac{\frac{1}{n}f_{\text{pre}}f_{\text{post}}\left(\tau_{+}\text{E}[A_{+}{\left(W\right)}^{n}]+\tau_{-}\text{E}[A_{-}{\left(W\right)}^{n}]\right)}{f_{\text{pre}}P_{l}^{fire}(W)\text{E}[A_{+}{\left(W\right)}^{n}]}\\ \;=\frac{1}{n}\left(\tau_{+}-\tau_{-}\frac{\text{E}[A_{-}{\left(W\right)}^{n}]}{\text{E}[A_{+}{\left(W\right)}^{n}]}\right)\frac{f_{\text{post}}}{P_{l}^{fire}(W)}\\ \;=\frac{1}{n}\left(\tau_{+}-\tau_{-}\frac{\text{E}[A_{-}{\left(W\right)}^{n}]}{\text{E}[A_{+}{\left(W\right)}^{n}]}\right)\\ \;\frac{f_{\text{pre}}\sum_{k}\frac{N_{c,k}}{m_{k}}\text{E}[P_{k}^{fire}(W)]}{P_{l}^{fire}(W)}. \end{array}$$

At a low pre-synaptic firing rate *f*_pre_, the dependency terms $M_{n,l}^{Dep}\left(W\right)$ gain influence and potentiate the synapses. At a high pre-synaptic firing rate *f*_pre_, the dependency terms $M_{n,l}^{Dep}\left(W\right)$ are relatively negligible and the synaptic weight distribution *P*(*W*) resembles that when the dependency terms $M_{n}^{Dep}\left(W\right)$ are ignored. In other words, a frequent pre-synaptic firing is more likely to induce a depression of other synapses. This is the mechanism by which the soft-bounded STDP model achieves the synaptic scaling. However, synapses potentiated by the dependency terms $M_{n}^{Dep}\left(W\right)$ lead to increase in post-synaptic firing rate *f*_post_, thereby reducing the influence of the dependency terms $M_{n}^{Dep}\left(W\right)$; thus, the soft-bounded STDP model cannot increase post-synaptic firing rate *f*_post_ sufficiently. This is one reason why the +NO and +LP models cannot prevent excessively low post-synaptic firing rates *f*_post_ as confirmed in Section 2.1. In contrast, intrinsic fluctuations regulate the synaptic weights *W* depending not on the dependency terms $M_{n}^{Dep}\left(W\right)$ but on the homeostatic terms $M_{n}^{H}\left(W\right)$. The homeostatic terms $M_{n}^{H}\left(W\right)$ with large values of parameters *S* and *s* can keep their influence after the post-synaptic firing rate *f*_post_ becomes larger; thus, the +IF model prevents the excessively low post-synaptic firing rate *f*_post_. In the +ADS model, when the post-synaptic firing rate *f*_post_ departs from the target *a*_*g*_, the homeostatic terms $M_{n}^{H}\left(W\right)$ gain influence and regulate the synaptic weights *W* to move *f*_post_ closer to *a*_*g*_.

The result theoretically-obtained from the +LP model with a high pre-synaptic firing rate *f*_pre_ = 20.0 is not consistent with the numerical result shown in Section 3.1.2 because of a factor not taken into account in the theoretical analysis. When an excitatory pre-synaptic firing with a strong weight is transmitted to the post-synaptic neuron, the membrane potential *v* gradually approaches the threshold voltage *v*_*th*_. If another excitatory pre-synaptic firing, which is not correlated to the former, is transmitted before the membrane potential *v* reaches the threshold voltage *v*_*th*_, it hastens the approach. Consequently, the synapses are more likely to be potentiated. This type of correlation between excitatory pre-synaptic firings gains influence when many synapses become strong and have high firing chances *P*^*fire*^(*W*); however, it is ignored in this theoretical analysis. For this reason, the +LP model leads to excessively high synaptic weights *W*. A more detailed analysis is outside the scope of this study.

#### 3.2.3. Competition

The synaptic weight distributions *P*_*l*_(*W*) of correlated groups *l* with different correlations *c*_*l*_ are also obtained from Equation (17). As is the case with Section 3.1.5, two groups are chosen and labeled *A* and *B*. Figure 7C shows the theoretical synaptic weight distributions *P*_*A*_(*W*) and *P*_*B*_(*W*) of the correlated groups *A* and *B*. According to Equation (20), the dependency terms $M_{n,l}^{Dep}\left(W\right)$ of the correlated group *l* lose their influence and the synapses are depressed when another correlated group *k* increases its firing chance $E\left[P_{k}^{fire}\left(W\right)\right]$. When the correlations are *c*_*A*_ = *c*_*B*_ = 0.04, the theoretical synaptic weight distributions *P*_*A*_(*W*) and *P*_*B*_(*W*) are the same. After the correlation for the correlated group *A* is changed to *c*_*A*_ = 0.16, the firing chance $P_{A}^{fire}\left(W\right)$ is increased, and the synapses in the correlated group *A* are potentiated. The responses of the correlated group *B* depend on the model. In the cases of the +NO and +LP models, the STDP terms $M_{n,B}^{STDP}\left(W\right)$ become dominant because of the increased post-synaptic firing rate *f*_post_; the dependency terms $M_{n,B}^{Dep}\left(W\right)$ lose their influence. The correlated group *B* loses a chance to induce a post-synaptic firing and to be potentiated. In the case of the +IF model, the STDP terms $M_{n,B}^{STDP}\left(W\right)$ become dominant, and the homeostatic terms $M_{n,B}^{H}\left(W\right)$ lose their influence. In addition to loss of a chance to be potentiated, the correlated group *B* is scaled down by soft-bounded STDP model because of increase in post-synaptic firing rate *f*_post_. In the case of the +ADS model, the activity-dependent scaling model reacts against the increasing post-synaptic firing rate *f*_post_ and the homeostatic terms $M_{n,B}^{H}\left(W\right)$ gain influence. Consequently, the synapses in the correlated group *B* are depressed. The three models exhibit competition, but their mechanisms differ. If a pre-synaptic firing rate *f*_pre_ is high, the dependency terms $M_{n,B}^{Dep}\left(W\right)$ have lost their influence, and the synaptic weight distribution *P*_*B*_(*W*) never changes in response to an increase in post-synaptic firing rate *f*_post_. This is why the +NO model with the pre-synaptic firing rate *f*_pre_ = 20 Hz did not exhibit competition in the previous studies (van Rossum et al., 2000; Gilson and Fukai, 2011). However, the +LP model does not exhibit competition in the numerical simulation as shown in Section 3.1.5 for the same reason as that for the excessive potentiation induced of the +LP model shown in the previous section.

## 4. Discussion

### 4.1. Stability of neuronal activity

Homeostatic plasticity called synaptic scaling is expected to maintain neuronal activity within a functional range by scaling up and down of synapses (Bienenstock et al., 1982; Turrigiano et al., 1998; Turrigiano, 1999, 2008; Burrone et al., 2002; Ibata et al., 2008; Keck et al., 2013; Vitureira and Goda, 2013; Zenke et al., 2013; Félix-Oliveira et al., 2014; Toyoizumi et al., 2014). Without homeostatic plasticity, the soft-bounded STDP model leaded to a post-synaptic firing rate of excessively low or high (see the left two panels of Figure 2). The soft-bounded STDP model prevents excessive change in synaptic weight; strong synapse is prone to be depressed and weak synapse is prone to be potentiated. Synaptic weights depart from upper and lower bounds and get closer to the equilibrium at which potentiation and depression are balanced with each other. The equilibrium depends on neuronal activity and the soft-bounded STDP model apparently achieves synaptic scaling by itself (see Figure 3A). However, with very low or very high pre-synaptic firing rate, synaptic weights at around the equilibrium lead to excessively high or low post-synaptic neuronal activity. The soft-bounded STDP model achieves at least stability of synaptic weight distribution but does not achieve stability of neuronal activity.

The soft-bounded STDP model incorporating intrinsic fluctuations (+IF model) maintained a post-synaptic firing rate within a certain range over a broad range of pre-synaptic firing rate. According to theoretical analyses, the contribution of intrinsic fluctuations is preventing excessively low neuronal activity by scaling up of synapses. Since the occurrence probability of STDP is roughly proportional to the product of the pre-synaptic and post-synaptic firing rates (van Rossum et al., 2000; Gilson and Fukai, 2011), with decreasing neuronal activity, the soft-bounded STDP model loses its influence and intrinsic fluctuations are relatively dominant; strong synapses are not depressed by the soft-bounded STDP model but rather diffused randomly by intrinsic fluctuations. Therefore, strong synapses stay strong and excessively low neuronal activity is prevented. This is the mechanism for scaling up achieved by intrinsic fluctuations. With increasing neuronal activity, the soft-bounded STDP model gains its influence and intrinsic fluctuations become negligible; strong synapses are depressed and thereby excessively high neuronal activity is prevented. This is the mechanism for scaling down. The soft-bounded STDP model also has the noise term $\nu_{p}\sim N\left(0,\sigma_{p}^{2}\right)$. It is almost negligible when compared with intrinsic fluctuations (see Figures 2, 3A) and also loses its influence with decreasing neuronal activity; the noise term ν_*p*_ does not contribute to stability of neuronal activity. Therefore, incorporation of intrinsic fluctuations and soft-bounded STDP model achieves scaling up and down of synapses and neuronal activity converges to the equilibrium at which activity-independent scaling up and activity-dependent scaling down are balanced with each other.

Intrinsic fluctuations contribute to competition between synapses but its mechanism differs from the others. When a group of excitatory synapses has an effect, another group reduces correlated activity and loses a chance to being potentiated. This is the mechanism of soft-bounded STDP model for synaptic competition (Song et al., 2000; van Rossum et al., 2000; Morrison et al., 2007). The activity-dependent scaling model reacts against increase in neuronal activity and depresses synapses as is the case with classical negative feedback processes (Bienenstock et al., 1982; Bear et al., 1987; Miller, 1996). On the other hand, intrinsic fluctuations do not react against increase in neuronal activity. Nonetheless increase in neuronal activity promotes soft-bounded STDP model, leading to scaling down of strong synapses which have been potentiated by intrinsic fluctuations. As a result, incorporation of intrinsic fluctuations and soft-bounded STDP model contribute to competition between synapses.

In the case of silenced neuronal activity, the soft-bounded STDP model cannot regulate synapses. Intrinsic fluctuations potentiate synapses slowly because soft-bounded Hebbian plasticity, which normally depresses strong synapses, is blocked. This result is consistent with homeostatic plasticity observed in biological experiments (Turrigiano et al., 1998; Ibata et al., 2008). After sufficiently long duration, the synaptic weight distribution converges to a power-law distribution with no well-defined mean. Nonetheless synaptic weights are kept within plausible values for several days. The activity-dependent scaling model potentiates synapses drastically, leading implausibly strong synapses within an hour. This is because neuronal activity falls below the target and never returns to the target, no matter how much activity-dependent scaling model potentiates synapses. Although both intrinsic fluctuations and the activity-dependent scaling model lose stability of synaptic weight distribution in the case of silenced neuronal activity, intrinsic fluctuations lead to more biologically plausible results. Intrinsic fluctuations derive a power-law distribution with an exponent equal to the degree in synaptic weight of their expression. When expression of intrinsic fluctuations has a term having degree greater than 2 in synaptic weight derives a power-law distribution with a well-defined mean. Examining such intrinsic fluctuations is a future work.

### 4.2. Stability of synapses

Even when the synaptic weight distribution is stable, it does not imply that the individual synapse is stable. Memory is considered to be stored as synapses connections, stability of synapse contributes to retention of memory. Intrinsic fluctuations however have a risk for disturbing memory retention since they are random fluctuations. This study has shown that intrinsic fluctuations have a slightly negative influence on the stability of strong synapses but it is almost negligible as long as the neuronal activity is not too small. Synapses are shuffled randomly not only by intrinsic fluctuations but also by STDP (see the lower part of Figure 1). A pre-synaptic firing has a chance to induce a post-synaptic firing leading to potentiation of the synapse and depression of another synapse. Frequent pre- and post-synaptic firings induce frequent occurrence of STDP, leading to shuffling of synapses. The influence of intrinsic fluctuations on stability of synapses is much smaller than that of STDP. A more detailed comparison is a future work.

Even after neuronal activity is silenced, intrinsic fluctuations shuffle synapses in contrast to the other models. Their negative influence on the stability of the strong synapses is no longer negligible when the neuronal activity is silenced. This result however does not imply instability of memory; intrinsic fluctuations adapt synapses to the environment that has changed.

### 4.3. Mechanism underlying intrinsic fluctuations

Several molecules and proteins have been considered to be associated with scaling up and down (Turrigiano, 2008). If intrinsic fluctuations are the mechanism underlying scaling up of synapses, they can be mediated by these factors. Another possible hypothesis is that these factors are associated with STDP but not with intrinsic fluctuations, and intrinsic fluctuations are just a noise in post-synaptic structure. Scaling up is not necessarily achieved by promotion of intrinsic fluctuations; it can also be achieved by disturbance of soft-bounded STDP (i.e., scaling down) as shown in Figure 5. In either case, examining effects of these factors on intrinsic fluctuations is a possible future work.

### 4.4. Conclusion

In summary, incorporation of intrinsic fluctuations and soft-bounded STDP model achieves scaling up and down of synapses and it is one plausible candidate mechanism for homeostatic plasticity called the synaptic scaling.

## Author contributions

TM designed research; TM and KU performed research and analyzed data; and TM wrote the paper.

### Conflict of interest statement

The authors declare that the research was conducted in the absence of any commercial or financial relationships that could be construed as a potential conflict of interest.

## Footnotes

^1^Another type of homeostatic plasticity, namely intrinsic plasticity, is also confirmed but is outside the scope of this paper (Desai et al., 1999; Turrigiano and Nelson, 2000; Abraham, 2008; Watt and Desai, 2010).

## Appendix

In this section, the synaptic weight distribution *P*(*W*) is derived taking into account the dependency of post-synaptic firing on pre-synaptic firings. The time courses of the membrane potential *v* and the conductance *g*_*E*_ are as follows:

(A1) $$\frac{d}{dt}\left(\begin{array}{c} v(t)\\ g_{E}(t) \end{array}\right)=\left(\begin{array}{cc} -1/\tau_{m} & -(v(t)-v_{E})R/\tau_{m}\\ 0 & -1/\tau_{g} \end{array}\right)\left(\begin{array}{c} v(t)-v_{L}\\ g_{E}(t) \end{array}\right).$$

A pre-synaptic firing with weight *W* at *t* = 0 immediately increases the conductance *g*_*E*_ by *W*. In this section, simplified condition is introduced in which the rise time and the rise amount of the membrane potential *v* in response to a pre-synaptic firing are assumed to be sufficiently small; thus, the decay of the membrane potential *v* from the initial value *v*(0) is ignored. Under this condition, the terms *v*(*t*) − *v*_*E*_ and *v*(*t*) − *v*_*L*_ are approximated by *v*(0) − *v*_*E*_ and *v*(*t*) − *v*(0), and Equation (A1) is solved as

(A2) $$v(t)-v_{0}=\frac{g_{0}(v_{E}-v_{0})R(e^{-t/\tau_{m}}-e^{-t/\tau_{g}})}{\tau_{m}/\tau_{g}-1},$$

where *v*_0_ = *v*(0) and *g*_0_ = *g*(0) = *W*. The membrane potential *v* reaches the maximum value at time

(A3) $$t_{\text{max}}=\frac{\log(\frac{\tau_{g}}{\tau_{m}})}{\tau_{m}^{-1}-\tau_{g}^{-1}}.$$

The excitatory post-synaptic potential (EPSP) Δ*v*_*E*_, which is the change in the membrane potential *v*, is

(A4) $$\Delta v_{E}(W,v_{0}):\;=v(t_{\text{max}})-v_{0}.$$

The inhibitory post-synaptic potential (IPSP) Δ*v*_*I*_, which is the change in the membrane potential *v* induced by an inhibitory pre-synaptic firing, is obtained in the same way. Immediately after the membrane potential *v* exceeds the threshold voltage *v*_*th*_, the post-synaptic neuron fires and potentiation is induced. The temporal difference Δ*t* between the pre-synaptic firing *t*_pre_ and the post-synaptic firing *t*_post_ is assumed to be approximately zero because of the simplified condition of the sufficiently small rise time. According to Equation (A2), a set of *m*_*l*_ synapses with weights **W** = {*W*_0_, …, *W*__*m*__*l*_−1_} stimulated at one time induces an EPSP of

(A5) $$\Delta v_{E}(W,v)=\sum_{i\;=\;0}^{m_{l}-1}\Delta v_{E}(W_{i},v),$$

because of the linear approximation. The chance $P_{l}^{fire}\left(\text{W}\right)$ that the correlated pre-synaptic firing induces a post-synaptic firing is

(A6) $$P_{l}^{fire}(W)=\int_{v_{min}(W)}^{v_{th}}P(v)dv,$$

where *v*_*min*_(**W**) is the minimal value of the membrane potential at which a post-synaptic neuron fires in response to the correlated pre-synaptic firing, i.e., *v*_*min*_ = min{*v*|*v* + Δ*v*_*E*_(**W**, *v*) ≥ *v*_*th*_}; *P*(*v*) is the probability distribution over time of the membrane potential *v*. The chance $P_{l}^{fire}\left(W\right)$ that an event at a synapse with weight *W* induces a post-synaptic firing is as follows:

(A7) $$\begin{array}{l} P_{l}^{fire}(W)=\text{ ​E}[P_{l}^{fire}(W)|W_{0}=W]\\ \;=\int \cdots \int P(W_{1})\cdots P(W_{m-1})P_{l}^{fire}(W)dW_{1}\cdots dW_{m-1} \end{array}$$

The probability distribution *P*(*v*) over time of the membrane potential is also derived by the Fokker-Planck equation. However, the membrane potential *v* is reset from *v*_*th*_ to *v*_*r*_. Taking into account this discontinuous phenomenon, the Fokker-Planck equation is as follows:

(A8) $$\frac{\partial }{\partial t}P(v,t)=-\frac{\partial }{\partial v}M_{1}(v)P(v,t)+\frac{1}{2}\frac{\partial^{2}}{\partial v^{2}}M_{2}(v)P(v,t)+I(v),$$

under the constraints that

(A9) $$P(v,t)=0\text{ for }v\geq v_{th},\int_{-\infty }^{v_{th}}P(v,t)dv=1,I(v)=\delta (v-v_{r})f_{\text{post}}.$$

The *n*-th moment M_*n*_(*v*) of the changes in the membrane potential *v* is

(A10) $$\begin{array}{l} M_{n}(v)=\int P(\Delta v)\Delta v^{n}d\Delta v\\ \;=\sum_{l}\frac{f_{pre}N_{c,l}}{m_{l}}\int P(W)\Delta v_{E}{\left(W,v\right)}^{n}dW\\ \;+f_{pre}N_{I}\Delta v_{I}{\left(W_{I},v\right)}^{n}\\ \;=\sum_{l}\frac{f_{pre}N_{c,l}}{m_{l}}\int \cdots \int P(W_{0})\cdots P(W_{m-1})\\ \;\Delta v_{E}{\left(\{W_{0},\ldots ,W_{m-1}\},v\right)}^{n}dW_{0}\cdots dW_{m-1}\\ \;+f_{pre}N_{I}\Delta v_{I}{\left(W_{I},v\right)}^{n}. \end{array}$$

Since the synaptic weight distribution *P*(*W*) and the probability distribution *P*(*v*) over time of the membrane potential *v* depend on each other, the Fokker-Planck equations in Equations (15) and (A8) are solved simultaneously.
